# Interferon-γ Induces Senescence in Normal Human Melanocytes

**DOI:** 10.1371/journal.pone.0093232

**Published:** 2014-03-28

**Authors:** Suiquan Wang, Miaoni Zhou, Fuquan Lin, Dongyin Liu, Weisong Hong, Liangjun Lu, Yiping Zhu, Aie Xu

**Affiliations:** 1 Department of Dermatology, Hangzhou Institute of Dermatology and Venereology, Third People's Hospital of Hangzhou, Hangzhou, Zhejiang Province, China; 2 Zhejiang Chinese Medical University, Hangzhou, Zhejiang Province, China; Columbia University Medical Center, United States of America

## Abstract

**Background:**

Interferon-γ (IFN-γ) plays an important role in the proceedings of vitiligo through recruiting lymphocytes to the lesional skin. However, the potential effects of IFN-γ on skin melanocytes and the subsequent contribution to the vitiligo pathogenesis are still unclear.

**Objective:**

To investigate the effects of IFN-γ on viability and cellular functions of melanocytes.

**Methods:**

Primary human melanocytes were treated with IFN-γ. Cell viability, apoptosis, cell cycle melanin content and intracellular reactive oxygen species (ROS) level were measured. mRNA expression was examined by real-time PCR. The release of interleukin 6 (IL-6) and heat shock protein 70 (HSP-70) was monitored by ELISA. β-galactosidase staining was utilized to evaluate melanocyte senescence.

**Results:**

Persistent IFN-γ treatment induced viability loss, apoptosis, cell cycle arrest and senescence in melanocytes. Melanocyte senescence was characterized as the changes in pigmentation and morphology, as well as the increase of β-galactosidase activity. Increase of p21^Cip1/Waf1^ protein was evident in melanocytes after IFN-γ treatment. IFN-γ induction of senescence was attenuated by siRNAs against p21, Janus kinase 2 (JAK2) or signal transducer and activator of transcription 1 (STAT1), but not by JAK1 siRNA nor by p53 inhibitor pifithrin-α. IFN-γ treatment increased the accumulation of intracellular ROS in melanocytes, while ROS scavenger N-acetyl cysteine (NAC) effectively inhibited IFN-γ induced p21 expression and melanocyte senescence. IL-6 and HSP-70 release was significantly induced by IFN-γ treatment, which was largely inhibited by NAC. The increase of IL-6 and HSP-70 release could also be observed in senescent melanocytes.

**Conclusion:**

IFN-γ can induce senescence in melanocytes and consequently enhance their immuno-competency, leading to a vitiligo-prone milieu.

## Introduction

The loss of melanocytes is the cause of skin de-pigmentation in vitiligo, an acquired disfiguring skin disorder which affects 0.5–1% of the worldwide population [1]. The pathogenesis of vitiligo is elusive but appears to involve immunologic factors, oxidative stress, sympathetic neurogenic disturbance or other factors [2]. Melanocyte-specific CD8+T lymphocytes-mediated autoimmune response is currently highlighted to be associated with the destruction of the melanocytes in vitiligo [3]–[5]. But the mechanisms that provoke the immune response against autologous melanocytes are still unclear. Since cytokines and the related inflammatory mediators modulate the activation and skin homing of lymphocytes [6], [7], they are the important research objectives for elucidating the onset of autoimmune vitiligo. On the other hand, the discovery of redox imbalance in the vitiligo links the oxidative stress to vitiligo [8]–[10]. Recently, melanocytes in non-lesional skin of vitiligo patients were further proven to display aberrant senescence-like features [10]. Vitiligo is accordingly proposed to be a degenerative disorder, possibly caused by continuous stress which leads to apoptosis or senescence in melanocytes [10]. Due to the fact that some cytokines also directly or indirectly regulate the proliferation and/or differentiation of melanocytes [11], it is possible that melanocyte degeneration and autoimmune response are triggered by the same spectrum of cytokines.

Studies have shown that various cytokines including interferon-γ (IFN-γ) [3], [12], tumor necrosis factor α (TNFα) [13], [14] and chemokine (C-C motif) ligand 22 (CCL22) [15] are differentially expressed in the lesional skin and serum of vitiligo patients and health controls, indicating their roles in vitiligo. Among these cytokines, IFN-γ becomes the most attractive molecule as a result of recent discoveries suggesting its critical roles in the onset and progression of autoimmune vitiligo [16], [17].

As a pro-inflammatory cytokine, IFN-γ is predominately released by Th1 lymphocytes, CD8+ cytotoxic T lymphocytes and NK cells [18]. Other cell types including antigen presenting cells, B cells and NKT cells can also secrete IFN-γ [19]–[21]. The presence of IFN-γ is important in early innate immune response against infection, whereas IFN-γ secretion by T lymphocytes displays complex effects on regulating adaptive immune response [22]. Aside from host defense, IFN-γ is implicated in pathology of some autoimmune diseases such as systemic lupus erythematosus, multiple sclerosis and insulin-dependent diabetes [23]. In terms of vitiligo, IFN-γ level was significantly increased in lesional and adjacent uninvolved skin, as well as in the serum of vitiligo patients [3], [12]. Recent studies using various mouse models of vitiligo confirmed that IFN-γ played an important role in skin de-pigmentation through inducing local accumulation of melanocyte-specific CD8+T cells [16], [17].

In this study, we aimed to understand the effects of IFN-γ on the viability and cellular functions of melanocytes, and the underlying mechanisms. Our data demonstrated that IFN-γ blocked cell cycle and induced senescence in melanocytes and consequently increased their immuno-competency by enhancing the expression of immune response accelerators including interleukin 6 (IL-6) and heat shock protein 70 (HSP-70). Our findings thus provide more evidence to support the critical roles of IFN-γ in the pathogenesis of vitiligo.

## Materials and Methods

### Ethics statement

Institutional Research Ethics Approval from Institutional Research Ethics Committee of Third Hospital of Hangzhou, Hangzhou, China was obtained to collect samples of human material for research. The Declaration of Helsinki Principles was followed and patients gave written informed consent.

### Cell culture

The primary normal melanocytes (NHM) were isolated from human foreskin specimens obtained after circumcision surgery. Vitiligo melanocytes (VHM) were isolated from normally pigmented skin in the gluteal regions of vitiligo patients. Cells were cultured in Hu16 medium (F12 supplemented with 10% fetal bovine serum (FBS), 20 ng/ml bFGF and 20 µg/ml IBMX). The cells were used between passages 2 and 5. Methods for the isolation and cultivation of melanocytes were described previously [24]. Melanocytes were seeded at a density of 1×10^4^ cells per well into 96-well plates or at a density of 3×10^5^ cells per well into 6-well plates and incubated overnight before experiments. All culture medium components were purchased from Life Technologies (NY, USA).

### Cell viability, cell cycle and apoptosis examinations

Cell viability was measured using a Non-Radioactive Cell Proliferation Assay kit (Promega, WI, USA) according to the manufacturer's protocol. The absorbance of the final reaction product was measured at 490 nm with a microplate spectrophotometer (SpectraMax190, Molecular Devices, CA, USA).

For cell cycle analysis, the melanocytes were fixed with pre-chilled 70% ethanol overnight at 4°C after the treatment. Prior to analysis, cells were spun down and re-suspended in staining solution (PBS with 50 µg/ml propidium iodide (PI) and 200 µg/ml RNase A). Cells were incubated at 37°C for 30 minutes and immediately assayed on a flow cytometer (FACScalibur, BD Biosciences, CA, USA).

Melanocyte apoptosis was detected with Annexin V-PI staining kit (Life Technoloiges). Cells were detached and re-suspended in 100 µl of binding buffer containing Annexin V-FITC and PI for 15 min at room temperature in the dark. Then, 400 µl of 1× binding buffer was added, and the cells were analyzed immediately with a flow cytometer.

### The melanin content measurement

Cell lysates were prepared by lysing melanocytes in 20 mM Tris-HCl (pH 7.2) containing 1% Triton X-100, 0.01% SDS, and a protease inhibitor cocktail (Roche Molecular Biochemical, IN, USA). Cell lysates were centrifuged at 12,000 rpm for 15 minutes at 4°C. Melanin in cell pellets was then dissolved in 1 N NaOH/10% DMSO by heating at 80°C for 1 h. The melanin content was assayed in a microplate spectrophotometer at 470 nm, and the relative melanin quantity was normalized with protein concentration of each sample which was measured by BCA protein assay kit (Beyotime, China).

### ELISA

Released HSP-70 was monitored using an HSP-70 high sensitivity enzyme linked immunosorbent assay (ELISA) kit (ENZO, Switzerland), and the release of IL-6 was measured using an IL-6 ELISA kit (Abcam, UK). Briefly, 100 µl of standards or the experimental supernatant were pipetted into microtiter plate and incubated for 2 hours at room temperature. After removal of the samples, the plate was washed and incubated with antibody specific for HSP-70 or IL-6, followed by incubation with secondary antibody conjugated to horseradish peroxidase. The plate was then incubated with substrate solution for 30 min before the reaction was terminated by the addition of stop solution. Optical density was read at 450 nm with a microplate spectrophotometer. HSP-70 or IL-6 concentration of each sample was converted from standard curve.

### Intracellular ROS measurement

Melanocytes were washed with PBS and incubated with 2 µM 2, 7-dichlorofluorescin diacetate (DCFH-DA) (Life Technologies) diluted in serum free medium at 37°C for 30 min. The intracellular ROS level was immediately analyzed with flow cytometer at an excitation wavelength of 488 nm and an emission wavelength of 530 nm.

### RNA Isolation and Real-time RT-PCR analysis

Total RNA was extracted from melanocytes with SV total RNA purification kit (Promega, Shanghai, China). Reverse transcript reaction was performed using QuantiTect Reverse Transcription Kit (Qiagen, Germany). Real time PCR was performed using QuantiFast SYBR Green PCR Kit (Qiagen). The expression levels of each gene was normalized against β-actin using the comparative C_t_ method, and expressed as percentage of control, with the control as 1. Sequences of primers are listed on the Table S1.

### RNA silencing

Melanocytes were transfected with siRNA pools for target genes and a non-targeting control siRNA (Genepharma, China) using Lipofectmine 2000 (Life Technologies) according to manufacturer's protocol. Cells were cultured for 48 hours before receiving further treatments.

### Immunoblotting

The proteins in the total cell lysates were separated by 10% SDS-PAGE followed by transferring to a nitrocellulose membrane. The membrane was blocked with 5% non-fat milk in TBST (50 mM Tris.Cl, pH 7.6, 150 mM NaCl, 0.1% Tween-20) for 1 hour at room temperature, followed by overnight incubation at 4°C with specific primary antibodies against p53, p21, p16, Jak1, Jak2, STAT1 and β-actin (Abcam, UK). The membrane was then washed with TBST and incubated with fluorescent dye-labeled secondary antibody for 1 h at room temperature in the dark. The protein immuno-complex was visualized by an Odyssey Infrared Imaging System (LI-COR, USA).

### Senescence Associated β-galactosidase (SA-β-gal) staining

The senescent status of the cells was detected using Senescence-galactosidase staining kit (Cell signaling technology, MA, USA). In brief, cells were washed with PBS and fixed with 1× fixation buffer for 5 min at room temperature. Cells were then washed three times with PBS and incubated with staining solution at 37°C for 24 hour. The reaction was stopped by washing off the staining solution. The percentage of SA-β-gal positive cells was determined after counting cells from five randomly selected fields. Representative fields were photographed at 10× objective.

### Statistical analysis

Student's t-test was used to assess statistical significance. A value of P<0.05 or P<0.01 was considered to be a significant difference. Data were expressed as the mean ± SD from at least three independent experiments.

## Results

### IFN-γ causes cell cycle arrest and apoptosis in normal human melanocytes

**Figure 1:**
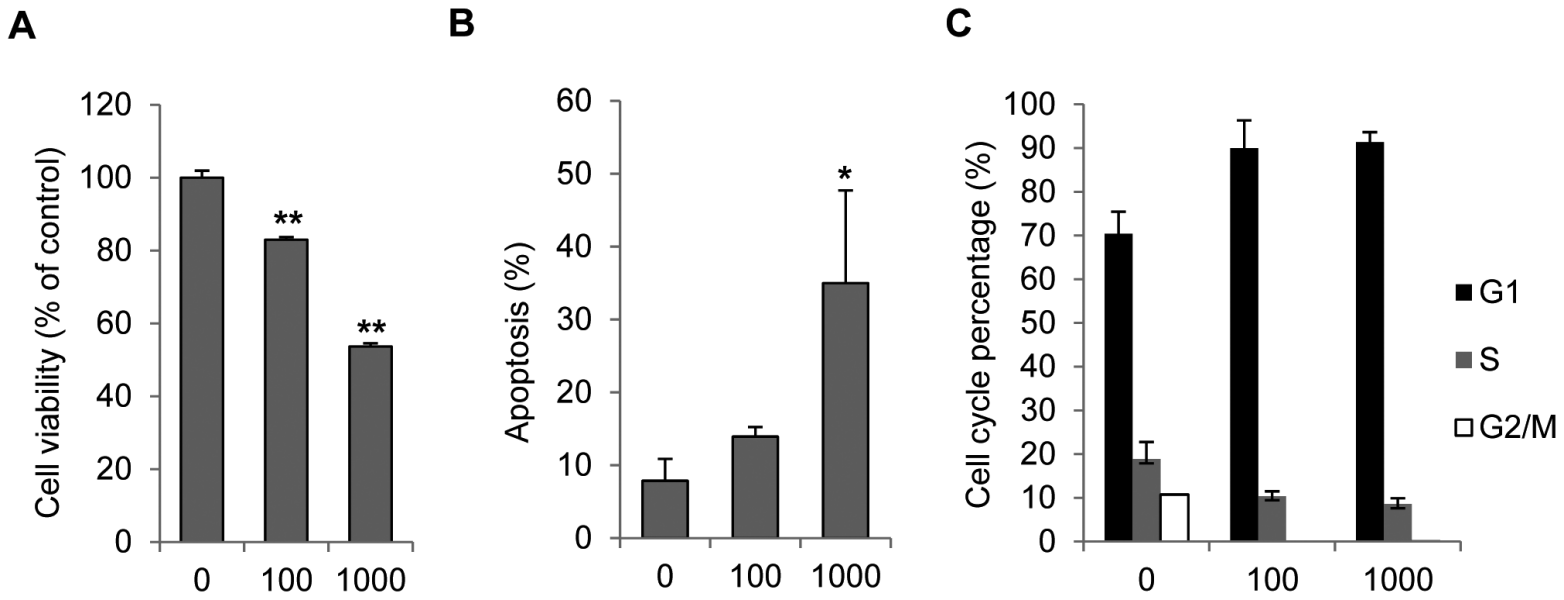
IFN-γ decreased viability of melanocytes, caused apoptosis and cell cycle arrest. Primary normal human Melanocytes were treated with various concentrations of IFN-γ (0, 100 or 1000 U/ml) for 72 h. Cell viability was then examined by MTS assay (A). Apoptosis was analyzed by flow cytometry after cells were stained with PI and Annexin V-FITC (B). (C) Cell cycle distribution of melanocytes was measured 24 h post IFN-γ treatment. Results are presented as mean ± SD from at least three independent melanocyte cultures. *P<0.05, **P<0.01, Student's t-test compared with controls.

To evaluate the effects of IFN-γ on melanocytes, we treated normal human melanocytes with IFN-γ and then examined the cell viability and apoptosis. As shown in Fig. 1A, IFN-γ significantly decreased the cell viability in a dose dependent manner. 1000 U/ml IFN-γ also caused obvious apoptosis in melanocytes (35.0% vs. 7.8% in untreated cells, p<0.05). In contrast, 100 U/ml IFN-γ induced very low rate of apoptosis (13.9% vs. 7.8%, Fig. 1B). Cell cycle analysis results demonstrated that IFN-γ at both concentrations caused the accumulation of melanocytes at G1 phase, while decreased the percentage of cells at S and G2/M phases (Fig. 1C). This result suggests that IFN-γ blocks cell growth by inducing G1/S cell cycle arrest.

### IFN-γ regulates the transcription of melanogenesis-related genes and increases melanin content in normal human melanocytes

**Figure 2:**
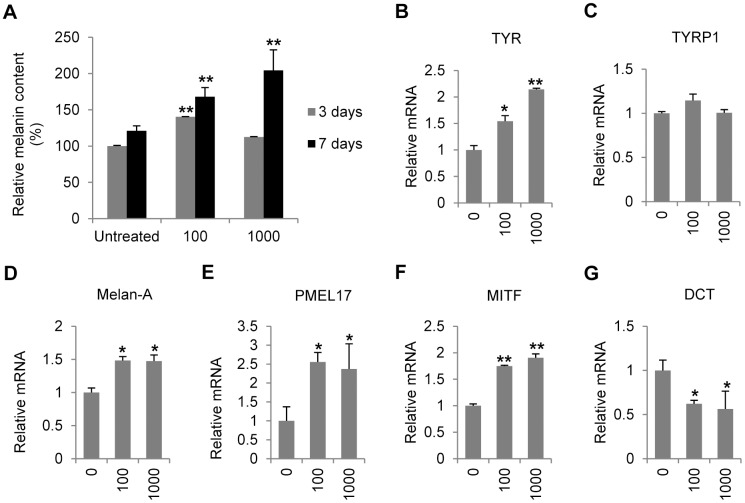
Effects of IFN-γ on melanogenesis in normal melanocytes. (A) Melanocytes were treated with various concentrations of IFN-γ (0, 100 or 1000 U/ml) for 3 or 7days before melanin content was measured. The melanin content was normalized on the basis of protein concentration. (b–g) Total RNA was extracted from melanocytes treated with or without IFN-γ for 24 hours. Real-time PCR was then performed to evaluate the relative mRNA levels of (B) tyrosinase (TYR), (C) tyrosinase-related protein 1 (TYRP1), (D) Melan-A, (E) melanocyte protein 17 (PMEL17), (F) microphthalmia-associated transcription factor (MITF), and (G) dopachrome tautomerase (DCT). The values shown represent the mean ± SD of three independent melanocyte cultures. *P<0.05 and **P<0.01.

To investigate the effect of IFN-γ on melanogenesis, melanocytes were treated with various concentrations of IFN-γ and harvested at 3 or 7 days after the treatment. The results demonstrated that 100 U/ml IFN-γ gradually and moderately increased the intracellular melanin level (40% on day 3 and 68% on day 7, p<0.01). 1000 U/ml IFN-γ didn't change the melanin level on day 3 but increased the melanin content on day 7 by 100% (Fig. 2A). We further examined the transcriptional profiles of melanogenesis-related genes. The results showed that IFN-γ up-regulated mRNA level of tyrosinase (TYR) (Fig. 2B), Melan-A (Fig. 2D), melanocyte protein 17 (PMEL17) (Fig. 2E) and microphthalmia-associated transcription factor (MITF) (Fig. 2F). IFN-γ had no significant effect on the mRNA expression of tyrosinase-related protein 1 (TYRP1) (Fig. 2C), but significantly decreased the transcription of dopachrome tautomerase (DCT) (Fig. 2G).

### IFN-γ induces senescence in melanocytes through p21^Cip1/Waf1^

**Figure 3:**
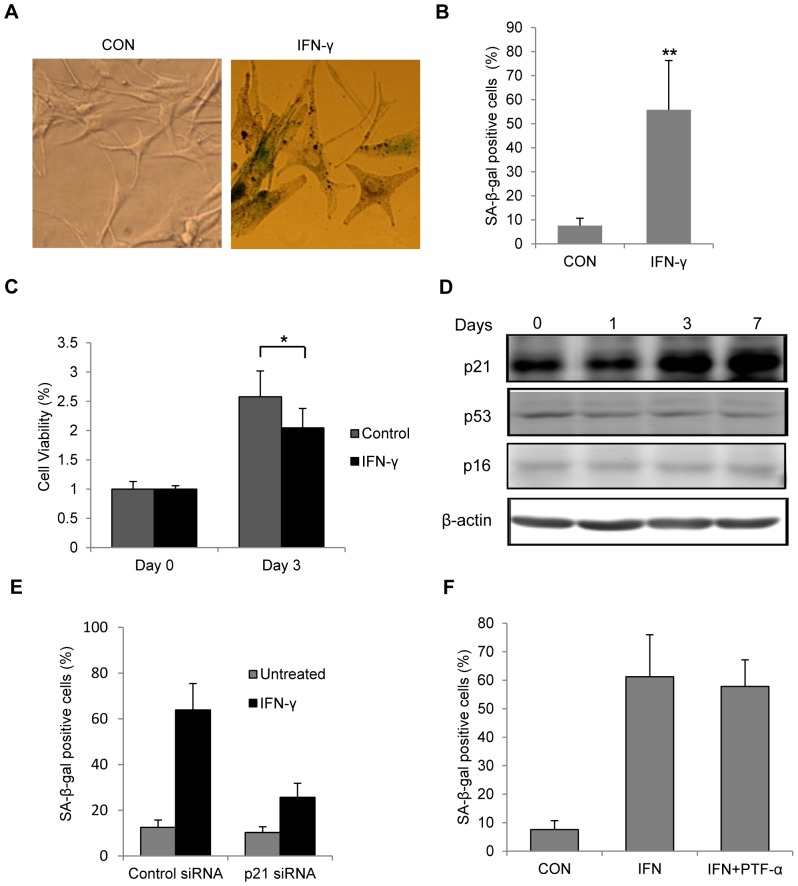
IFN-γ caused senescence in melanocytes through p21 pathway. Melanocytes were treated with or without 100/ml IFN-γ for 7 days. Senescence was evaluated based on SA-β-gal staining, and cell morphology. (A) Representative pictures of SA-β-gal-stained cells observed under bright-field microscope. Flattened and enlarged cells with blue/green stain were regarded as senescent cells (B) Quantification of SA-β-gal-positive cells based on microscopic analysis. CON represents the control cells. **P<0.01. (C) After 7 days of treatment, melanocytes were cultured in fresh medium without IFN-γ for 3 days and cell viability was examined by MTS assay. (D) Melanocytes were cultured in the presence or absence of IFN-γ for up to 7 days and cells were harvest on day 1, 3 and 7. Cell lysates were subjected to SDS-PAGE and analyzed by western blot with indicated antibodies. β-actin was probed as the loading control. (E,) Bar graphs of SA-β-gal staining results. (E) Melanocytes were transfected with scrambled control or p21 siRNAs for 48 h before IFN-γ treatment. (F) Melanocytes were treated with or without 100 U/ml IFN-γ for 7 days in the presence of DMSO or 20 µM pifithrin-α (PFT-α).

The morphological pictures showed that the normal adult human melanocytes were pale, dendritic with small cell bodies. In contrast, melanocytes after persistent IFN-γ treatment became large, flat in shape with shorter and fewer dendrites, and some cells were highly pigmented (Fig. 3A). We also noticed a significant increase of SA-β-gal staining, a marker of senescence, in melanocytes with 7 days of IFN-γ stimulation (Fig. 3A, B). IFN-γ treated melanocytes grew slower than untreated normal cells even after the removal of IFN-γ (Fig. 3C). p53/p21^Cip1/Waf1^ and p16^INK4a^ are two major pathways that mediate senescence [25], [26]. Depending on the cell type or stressor, senescence might be mediated by activation of either of the pathway [26]–[28]. Immunoblotting analysis indicated that protein level of p21 was greatly elevated with the increasing duration of IFN-γ treatment, while p16 level didn't change during the experiment (Fig. 3D). Surprisingly, the protein level of p53, the transcriptional activator of p21, didn't show significant increase upon IFN-γ treatment (Fig. 3D). In vitiligo melanocytes, IFN-γ treatment also increased the p21 protein level accompanied by the increase of SA-β-gal expression without changing the protein levels of p53 and p16 (Fig. S1). To verify whether p21 was required for the melanocyte senescence induced by IFN-γ, we transfected melanocytes with siRNA pools targeting p21. As demonstrated, p21 siRNA treatment suppressed the IFN-γ-induced increase of SA-β-gal staining (Fig. 3E), while p53 inhibitor pifithrin-α failed to have such an effect (Fig. 3F).

### IFN-γ-induced p21 expression and senescence depend on JAK2 and STAT1 signaling in melanocytes

**Figure 4:**
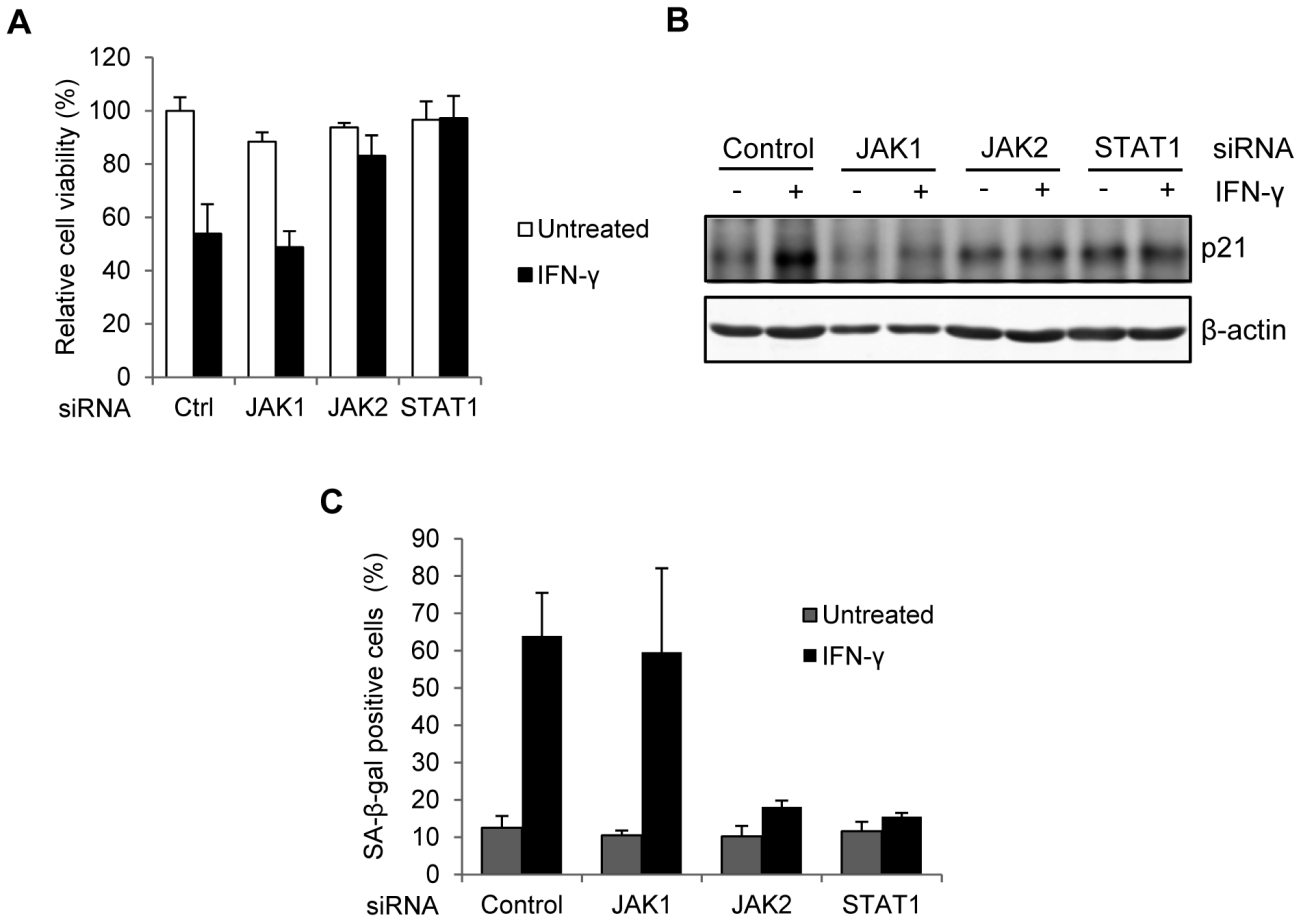
JAK2 and STAT1 activities are necessary for IFN-γ caused melanocyte senescence. Melanocytes were transfected with JAK1, JAK2, STAT1 siRNAs or scrambled control siRNA (Ctrl). After 48 h, cells were treated with or without 100 U/ml IFN-γ for additional 7 days. (A) Cell viability of melancytes was measured by MTS assay. (B) Protein level of p21 was evaluated by Western blot. β-actin was probed as the loading control. (C) Percentages of SA-β-gal-positive cells were determined based on microscopic analysis.

In canonical IFN-γ signaling, IFN-γ bound receptor complex recruits Janus kinase 1 (JAK1) and JAK2 kinases, leading to the phosphorylation and nuclear translocation of signal transducer and activator of transcription 1 (STAT1), which in turn transcriptionally activates downstream IFN-γ inducible genes [18]. To elucidate the possible involvement of JAK/STAT signaling in IFN-γ induced melanocyte senescence, we transfected siRNA pools into melanocytes to knock down the mRNA expression of JAK1, JAK2 and STAT1 respectively. In the presence of IFN-γ, the cell viability of control siRNA transfected melanocytes was significantly inhibited (Fig. 4A). Significantly, JAK2 or STAT1 siRNA efficiently restored the viability of melanocytes, whereas JAK1 siRNA didn't have such an effect (Fig. 4A). Immunoblotting results confirmed that JAK2 or STAT1 siRNA, but not JAK1 siRNA inhibited the increase of p21 induced by IFN-γ (Fig. 4B). Moreover, IFN-γ-induced SA-β-gal staining increase in melanocytes was blocked by JAK2 and STAT1 siRNAs (Fig. 4C). Thus, IFN-γ-induced p21 expression and senescence depend on JAK2 and STAT1 signaling in melanocytes

### IFN-γ-induced melanocyte senescence requires reactive oxygen species (ROS)

**Figure 5:**
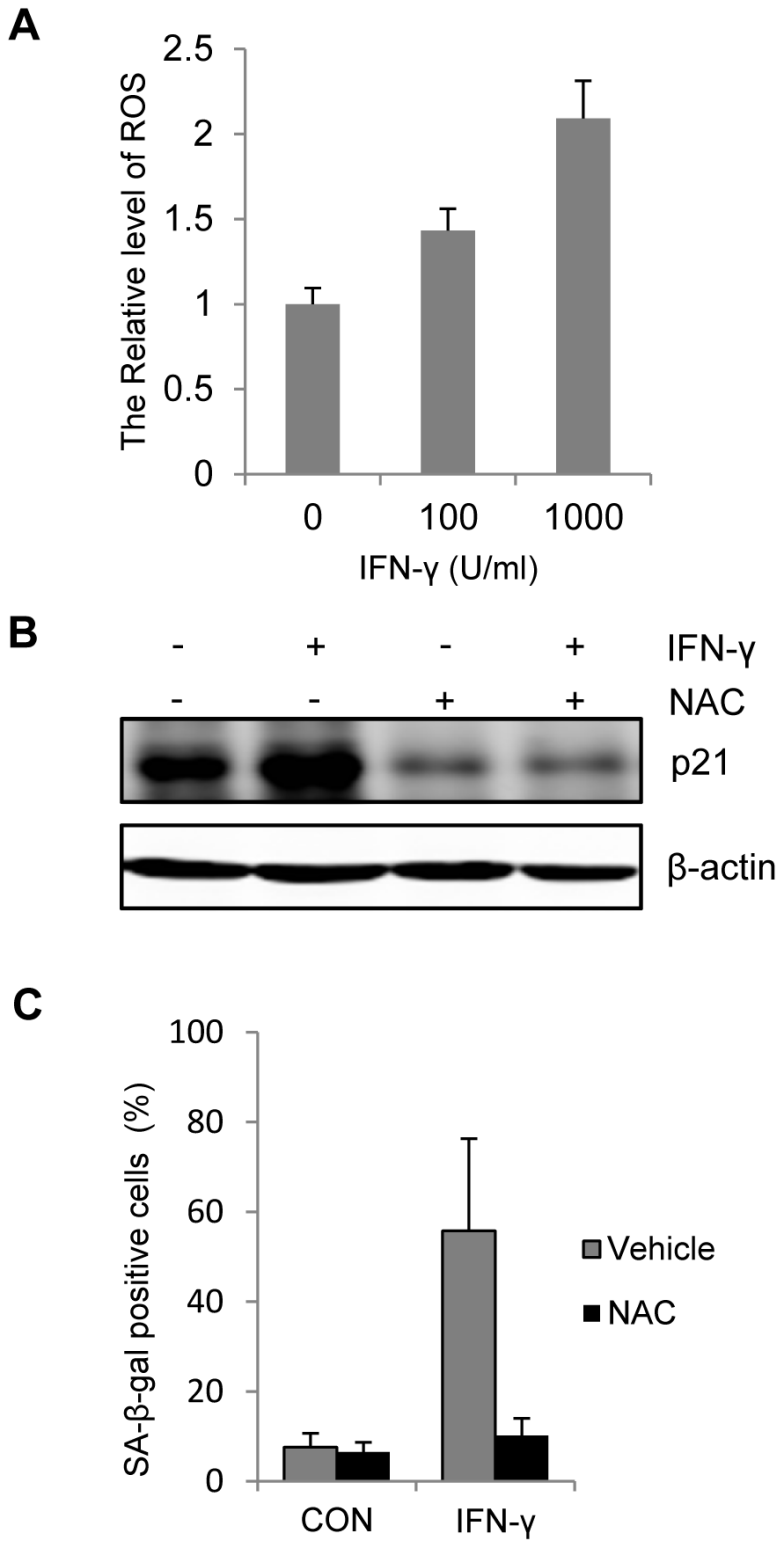
Involvement of Reactive Oxygen Species (ROS) in the IFN-γ induced senescence. (A) Melancytes were stimulated with indicated concentration of IFN-γ for 24 h. Generated ROS was detected with flow cytometer after labelled with the ROS sensor DCFH-DA. (B,C) Melanocytes were treated with or without 100 U/ml IFN-γ for 7 days in the presence of vehicle or 1 mM NAC. (B) Protein level of p21 was evaluated by Western blot. β-actin was probed as the loading control. (C) Percentages of SA-β-gal-positive cells were determined based on microscopic analysis. CON represents the cell culture without IFN-γ.

ROS has been reported to participate in the senescence induction [29]–[31]. As demonstrated in Fig. 5A, IFN-γ treatment caused obvious elevation of intracellular ROS, and the effect of IFN-γ was dose-dependent manner. To verify the contribution of ROS to IFN-γ induced senescence, we supplied N-acetyl cysteine (NAC) to the medium as the ROS scavenger. Addition of NAC inhibited the increase of p21 protein level induced by IFN-γ (Fig. 5B). Meanwhile, NAC effectively prevented the increase of SA-β-gal positive cells with IFN-γ treatment (Fig. 5C). Thus, ROS is also involved in IFN-γ-induced p21 expression and melanocyte senescence.

### ROS and senescence status enhanced the secretion of IL-6 and HSP-70 from melanocytes

**Figure 6:**
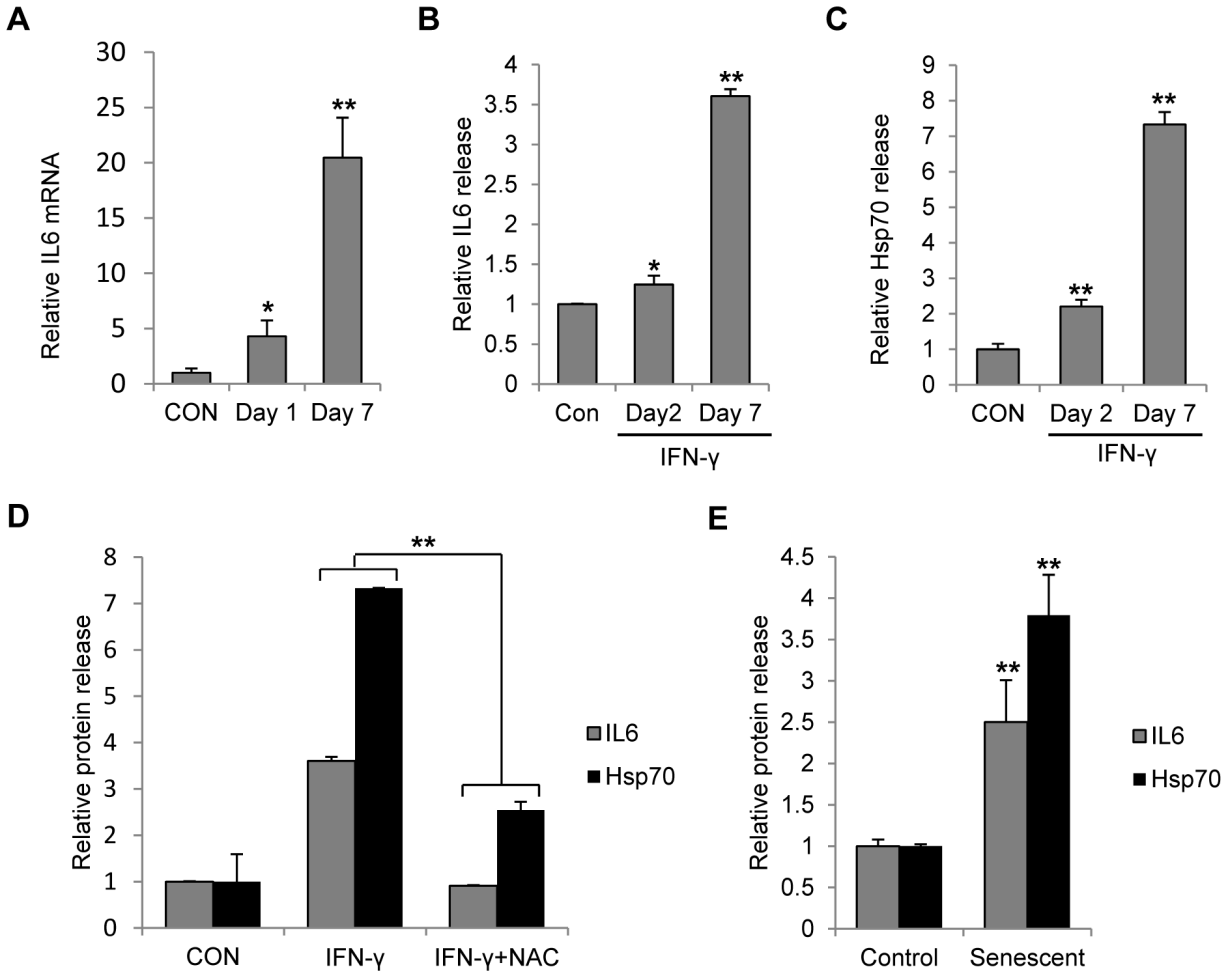
Release of IL-6 and hsp70 from melanocytes was enhanced after persistent IFN-γ treatment or senescence induction. (A) Melanocytes were treated with or without 100 U/ml IFN-γ for continuous 7 days. RNA was extracted from melanocytes at day 1 and day 7. Real time PCR was performed to evaluate the transcription of IL-6 in these cells. CON represents the control cells. (B–D) Melanocytes were treated with or without 100 U/ml IFN-γ for 7 days in the presence of vehicle or 1 mM NAC. Supernatants of cell culture were collected at the indicated time points. Medium was changed 48 h before the supernatant collection. Release of IL-6 (B) or HSP-70 (C) in response to IFN-γ treatment was monitored by ELISA analysis. (D) The effect of NAC on the release of IL-6 and HSP-70 after IFN-γ treatment was evaluated. (E) Melanocytes were treated with or without 100 U/ml IFN-γ for 7 days to induce senescence. Senescent melanocytes were then cultured in normal medium for 4 days before the supernatant was collected. The medium was changed 48 h before the supernatant collection. Protein levels of released IL-6 and HSP-70 from senescent cells were compared with those from normal cells.

Normal melanocytes produce various cytokines and other immune-related factors [32], [33]. We first measured the mRNA level of IL-6 in the melanocytes exposed to IFN-γ. It was shown that 24 hour of IFN-γ stimulation significantly enhanced the IL-6 transcription by about 4-fold. When the IFN-γ duration prolonged to 7 days, the enhancement of IL-6 transcription was increased even higher to 20-fold (Fig. 6A). The ELISA results confirmed time-dependent IL-6 secretion in melanocytes after IFN-γ treatment (Fig. 6B). Similar to IL-6, the secretion of heat shock protein 70 (HSP-70) was also enhanced significantly with IFN-γ stimulation (Fig. 6C). In addition, the effect of IFN-γ on IL-6 and HSP-70 was largely inhibited by the ROS scavenger NAC (Fig. 6D). To determine whether the senescence status changes the release of IL6 and Hsp70, we collected the supernatants from IFN-γ-induced senescent melanocytes and normal melanocytes. The result indicated that senescent melanocytes released significantly higher amount of IL-6 and HSP-70 compared with normal melanocytes (Fig. 6E). Thus, IFN-γ-induced IL-6 and HSP-70 release was associated with ROS production and cell senescence.

## Discussion

Autoimmunity and oxidative stress are considered as key factors involved in the destruction of melanocytes in vitiligo. Despite the existence of accumulating evidence supporting the pathogenic role of oxidative stress, there is a lack of convincing proof indicating the occurrence of cytotoxicity or apoptosis in vitiligo skin *in vivo* [33]. Previous studies suggested that melanocytes in non-lesional skin of vitiligo patients displayed aberrant features [10], [35], [36], including sensitive to oxidative stress, easy to detach after skin friction and increased production of biologically active proteins among the senescence-associated secretory phenotype (SAPS), such as IL-6 and matrix metalloproteinase-3, compared with melanocytes from normal healthy controls. It is then proposed that vitiligo is a degenerative disease with melanocytes showing pre-senescence phenotype caused by oxidative and other stresses [10]. In this study, we observed that persistent exposure to IFN-γ caused melanocyte senescence for which ROS is required. Because IFN-γ is present in various inflammatory conditions and is found to be elevated in the vitiliginous skin, it is possible that depigmentation in vitiligo arises from localized inflammation, where IFN-γ interferes with the cell viability of surrounding melanocytes, leading to the senescence-driven melanocyte detachment.

Melanocyte senescence is often accompanied with the increase of pigmentation [37], [38]. In our study, IFN-γ promoted the accumulation of melanin in melanocytes, and increased the transcription of some of the melanogenesis-related genes. However, it also significantly decreased the mRNA level of DCT, which encodes a critical enzyme in the synthesis of eumelanin [39]. Thus, IFN-γ might change the eumelanin/pheomelanin ratio in melanocytes. Even though IFN-γ treatment increases the melanin content in melanocytes, it also causes the morphologic changes of melanocytes including shortened dendrites which might be associated with the change of their melanosome transferring capacity [40]. Therefore, the overall impact of IFN-γ and melanocyte senescence on skin pigmentation is needed to be determined by further study.

p53/p21 and p16 are two main pathways engaged in the regulation of senescence [25], [26]. In a stress condition, p53 might be activated and it in turn transcriptionally activates p21 to execute the senescence induction. However, p21 can also be induced in a p53-independent way [41], [42]. Previous study demonstrated the main contribution of p16 on accelerated senescence observed in vitiligo melanocytes [10] or senescence in normal melanocytes at high passage levels [43]. Here we demonstrated that IFN-γ treatment affected the expression of p21 in both of normal melanocytes and vitiligo melanocytes without changing the protein levels of p53 and p16, and only knocking-down of p21 was effective to prevent the IFN-γ-induced melanocyte senescence. These results suggest that IFN-γ induced melanocyte senescence is mediated by p21, but not by p53 or p16.

Among the factors that can be released from melanocytes, we are particularly interested in IL-6. Part of reason is that IL-6 is an important immune reaction regulator and considered to play critical roles in the pathogenesis of various autoimmune disorders [44]–[46]. Increasing serum and/or lesional skin levels of IL-6 have been documented in vitiligo [47]. Additionally, it has been shown that IL-6 directly inhibits the growth and modulates antigen expression of melanocytes [48]. High level of IL-6 in the lesional skin of vitiligo was indicated to be relevant to the failure of melanocyte transplantation therapy (unpublished data). It was hypothesized that increasing IL-6 links melanocyte stress and immune targeting of these cells [32]. Another molecule that plays important roles in vitiligo is HSP-70. HSP-70 is a molecular chaperon which protects cellular proteins from premature degradation by supporting proper protein folding. It can be released into extracellular environment. In contrast to the cytoprotective function of intracellular HSP-70, extracellular HSP-70 is immunogenic and associated with some autoimmune disease [49], [50]. HSP-70 has recently gained the attention as a critical molecule to accelerate immune response against melanocytes in vitiligo [51], [52]. Secreted HSP-70 from melanocytes can activate dendritic cells (DCs) and enhance the capacity of DCs to uptake and presenting antigens leading to an increased vitiligo response [52], [53]. Whereas, an HSP-70 molecule with single mutation in the DC binding region interferes with the activation of DC and reverses the depigmentation in a mouse vitiligo model [54]. In this study, we found that release of IL-6 or HSP-70 by melanocytes was significantly elevated upon persistent exposure to IFN-γ or after senescence induction. These results indicate that IFN-γ can profoundly affect the immuno-competency of melanocytes.

Redox imbalance occurs generally in vitiligo [34]. H_2_O_2_, a main source of ROS, has been proven to accumulate in the epidermis of acute vitiligo patients [9]. The findings of ROS involvement in the IFN-γ induced melanocyte senescence and stimulation of IL-6 and HSP-70 release provides new evidence of link among oxidative stress, melanocyte degeneration and autoimmune vitiligo [55]. It is conceivable to hypothesize that increased ROS by other factors may also trigger melanocyte senescence, and the following release of IL-6 and HSP-70 as well. If the hypothesis is correct, it will give us an additional rationale to use antioxidants for the treatment of vitiligo.

Taken together, our findings support the idea that IFN-γ directly decreases the viability of melanocytes, and meanwhile it helps create a vitiligo-prone milieu by enhancing the release of some autoimmune accelerators such as IL-6 and HSP-70. The overall effects then facilitate the onset and progress of vitiligo. More studies are necessary to be carried out to further elucidate the mechanism and impact of melanocyte senescence to develop effective strategies on vitiligo treatment.

## Supporting Information

Figure S1 **Analysis of senescence-related gene expression in vitiligo melanocytes after IFN-γ treatment.** Vitiligo melanocytes (V1–V3) and normal melanocytes (NHM) were treated with or without IFN-γ for 7 days. (A) Cell lysates were subjected to SDS-PAGE and analyzed by western blot with indicated antibodies. β-actin was probed as the loading control. (B) SA-β-gal expression in vitiligo melanocytes (VHM) or normal melanocytes (NHM) was determined based on microscopic analysis.
(TIF)

Table S1 **List of primers for real-time PCR reaction.**
(DOCX)
