# Supplementary figures and images for: Interferon-γ Induces Senescence in Normal Human Melanocytes
Source: PLoS One. 2014 Mar 28;9(3):e93232. doi: 10.1371/journal.pone.0093232 (PMC3969336; doi:10.1371/journal.pone.0093232)

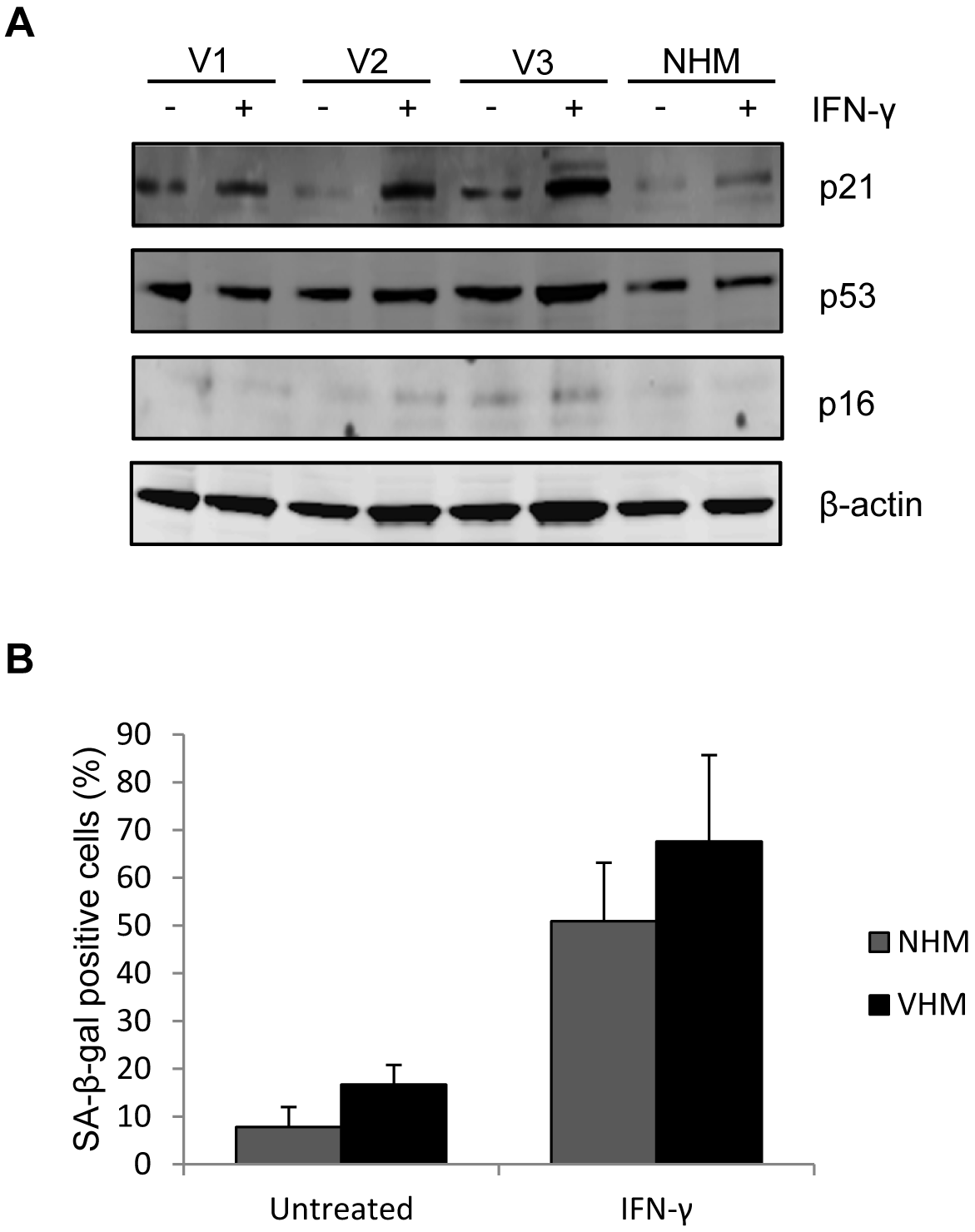

Supplement: Figure S1 — Analysis of senescence-related gene expression in vitiligo melanocytes after IFN-γ treatment. Vitiligo melanocytes (V1–V3) and normal melanocytes (NHM) were treated with or without IFN-γ for 7 days. (A) Cell lysates were subjected to SDS-PAGE and analyzed by western blot with indicated antibodies. β-actin was probed as the loading control. (B) SA-β-gal expression in vitiligo melanocytes (VHM) or normal melanocytes (NHM) was determined based on microscopic analysis. (TIF) [file pone.0093232.s001.tif]
