# Supplementary material for: Interferon-γ Induces Senescence in Normal Human Melanocytes
Source: PLoS One. 2014 Mar 28;9(3):e93232. doi: 10.1371/journal.pone.0093232 (PMC3969336; doi:10.1371/journal.pone.0093232)
Supplement: Table S1 — List of primers for real-time PCR reaction. (DOCX) [file pone.0093232.s002.docx]

Table S1 List of primers for real-time PCR reaction

Genes Forward primers Reverse primers

β-actin 5’-ATAGCACAGCCTGGATAGCAACGTAC-3’ 5’-CACCTTCTACAATGAGCTGCGTGTG-3’

TYR 5’-TGCACAGAGAGACGACTCTTG-3’ 5’-GAGCTGATGGTATGCTTTGCTAA-3’

TYRP1 5’-TCTCTGGGCTGTATCTTCTTCC-3’ 5’-GTCTGGGCAACACATACCACT-3’

Melan-A 5’-GCTCACTTCATCTATGGTTACCC-3’ 5’-GACTCCCAGGATCACTGTCAG-3’

PMEL17 5’-AGGTGCCTTTCTCCGTGAG-3’ 5’-AGCTTCAGCCAGATAGCCACT-3’

MITF 5’-GCCTCCAAGCCTCCGATAAG-3’ 5’-GCACTCTCTGTTGCATGAACT-3’

DCT 5’-AACTGCGAGCGGAAGAAACC-3’ 5’-CGTAGTCGGGGTGTACTCTCT-3’

IL6 5’-ACTCACCTCTTCAGAACGAATTG-3’ 5’-CCATCTTTGGAAGGTTCAGGTTG-3’
